# Asymmetric Membranes Obtained from Sulfonated HIPS Waste with Potential Application in Wastewater Treatment

**DOI:** 10.3390/membranes14120247

**Published:** 2024-11-22

**Authors:** Marcial Alfredo Yam-Cervantes, Rita Sulub-Sulub, Mauricio Hunh-Ibarra, Santiago Duarte, Erik Uc-Fernandez, Daniel Pérez-Canales, Manuel Aguilar-Vega, Maria Ortencia González-Díaz

**Affiliations:** 1 Centro de Investigación Científica de Yucatán, A.C., Calle 43 No. 130, Chuburná de Hidalgo, Mérida 97200, Yucatán, Mexicomjav@cicy.mx (M.A.-V.); 2 Independent Researcher, Calle Florida 9 Int. 17, Col. Noche Buena, Del. Benito Juárez, Ciudad de México 03720, Distrito Federal, Mexico; 3 CONAHCYT—Centro de Investigación Científica de Yucatán, A.C., Calle 43 No. 130, Chuburná de Hidalgo, Mérida 97200, Yucatán, Mexico

**Keywords:** asymmetric membranes, high-impact polystyrene waste, dye removal, water treatment, sulfonated high-impact polystyrene

## Abstract

The recovery and reuse of high-impact polystyrene (HIPS) into high-value products is crucial for reducing environmental thermoplastics waste and promoting sustainable materials for various applications. In this study, asymmetric membranes obtained from sulfonated HIPS waste were used for salt and dye removals. The incorporation of sulfonic acid (-SO_3_H) groups into HIPS waste by direct chemical sulfonation with chlorosulfonic acid (CSA), at two different concentrations, was investigated to impart antifouling properties in membranes for water treatment. Asymmetric membranes from recycled HIPS, R-HIPS, R-HIPS-3, and R-HIPS-5 with 3 and 5% sulfonation degrees, respectively. Sulfonated HIPS shows a decrease in water contact angle (WCA) from 83.8° for recycled R-HIPS to 66.1° for R-HIPS-5, respectively. A WCA decrease leads to an increase in antifouling properties for R-HIPS-5, compared to non-sulfonated R-HIPS, which leads to a higher flux recovery ratio (FRR) and enhanced separation properties for sulfonated membranes. The HIPS-5 membrane exhibited the highest rejection rates for Reactive Black 5 dye (94%) and divalent salts (72% for MgSO_4_ and 67% for Na_2_SO_4_). The performance of the recycled HIPS asymmetric membranes is well correlated with porosity, water uptake, and the higher negative charge from the sulfonic acid groups present, which enhance the electrostatic repulsions of salts and dyes.

## 1. Introduction

In recent years, there has been a substantial rise in global plastic waste, leading to serious environmental and public health challenges [1]. In 2018, annual plastic production reached 359 million tons, and it is expected to double by 2050. In Mexico, the per capita plastic waste generation is 59 kg annually, with less than 10% being recycled [2]. In 2023, the Mexican Secretariat of Environment and Natural Resources (SEMARNAT) identified polypropylene (PP), low-density polyethylene (LDPE), and polystyrene (PS) as the primary polymers contributing to environmental pollution, with PS having the lowest recycling potential based on a cost–benefit ratio [2]. In response, the Mexican government, following international initiatives, proposed a policy for sustainable development and a circular economy to enhance waste management practices. A key initiative within this framework is the redesign and reuse of plastic products. Specifically, recycling and transforming polystyrene—available in several forms, such as expanded polystyrene (EPS), general-purpose polystyrene (GPPS), and high-impact polystyrene (HIPS)—is crucial for reducing PS waste in the environment and promoting sustainable materials for various applications.

Recently, single-use polystyrene (PS) waste has been identified as a valuable material for the circular economy [3,4,5]. For example, nanofiber membranes from expanded polystyrene (EPS) waste with different poly(vinylpyrrolidone) (PVP) concentrations were elaborated by electrospinning for their use in water filtration [6]. PVP introduction reduced EPS’s high hydrophobicity on the membrane surface, changing their water contact angle from 140.46° to 84.70° and increasing pure water flux from 526.86 L m^−2^h^−1^ to 1901.35 L m^−2^h^−1^. The introduction of carbon nanotubes is also a strategy to obtain a more hydrophilic membrane surface with lower fouling properties [7]. Aquino Lima et al. developed an ultra-fast method to produce recycled EPS membranes using DL-limonene as a natural solvent, with adequate characteristics for its applications in air filtration [8]. Moreover, the production of ion exchange resins from polystyrene waste, sulfonated with sulfuric acid, has been reported for heavy metals (Cu^2+,^ Zn^2+^, and Cd^2+^) removal in wastewater [9,10,11], as well as for the adsorption of fluoroquinolone, a second-generation synthetic antibiotic [12]. Membranes from sulfonated PSE blended with 1% of polybutylene succinate (PBS) exhibited a higher methylene blue uptake and good regeneration efficiency, compared with membranes containing a higher PBS amount, following a pseudo-second order kinetic model in the adsorption process [13].

The utilization of HIPS waste has been less extensively studied, despite its notable advantages over brittle and rigid EPS and GPPS [14,15]. HIPS offers superior impact resistance, flexibility, and stability, making it a promising candidate for various applications. Sharma et al. investigated the potential use of recycled HIPS as a partial replacement for fine aggregates in lightweight concrete and evaluated the impact on key properties, such as compressive strength, flexural strength, and bond strength. The authors concluded that recycled HIPS at 10% concentration is an optimal level for achieving lightweight concrete with desirable mechanical properties, offering a sustainable solution for recycling plastic waste in construction materials [16]. Moreover, cation exchange resins for the removal of heavy metal ions (Zn^2+^, Pb^2+^) were prepared from sulfonated polystyrene waste (WPS) containing HIPS and EPS. These cation exchange resins were capable of reducing Zn^2+^ metal concentration from 80 mg/L to 38.3 mg/L and Pb^2+^ from 100 mg/L to 33 mg/L by an absorption process [17]. Air filter membranes were also fabricated from a HIPS waste solution in a D-limonene:DMF mixture using needleless electrospinning. A fine fiber morphology was achieved by increasing DMF concentration in the solvent mixture. The HIPS membrane demonstrated excellent performance as an air filtration medium, with a filtration efficiency up to 98.5%, as shown in filtration tests with PM_2.5_ particles [18]. Khaled et al. elaborated micro-porous membranes from recycled HIPS, which presented contact angle values between 81° and 91°, with surface pore sizes ranging from 150 nm to 550 nm and with macrovoids in their cross-sections [19]. These membranes presented Rhodamine B (RhB) rejection values between 95 and 97% and from 85 to 87% for humic acid (HA) [20]. They exhibited a slight increase in tensile strength values as HIPS concentration increased from 20 to 35% [15]. Waste HIPS’s feasibility as membranes for gas separation was also investigated [21]. The authors reported that HIPS membranes exhibited better gas transport properties than EPS and GPPS, due to their butadiene rubber content in the matrix, with a gas separation factor that was well correlated with the difference in the gas penetrant kinetic diameter. Recently, a method for the total revalorization of HIPS was investigated by Giakoumakis et al. These authors employed HIPS waste as potential feedstock for styrene monomer recovery using a green fractionation process using ethyl acetate (EtOAc). This strategy consists of EtOAc addition as a pre-fractionation solvent to isolate the free PS matrix from the rubber phase, followed by thermal degradation of the isolated free PS matrix, which leads to an enhanced styrene yield, compared to the non-fractionated HIPS sample, and finally, an ethylene metathesis for the valorization of the rubber phase [22].

Here, we present the development of asymmetric sulfonated HIPS membranes from recycled HIPS (R-HIPS) for water treatment. The incorporation of sulfonic pendant groups in R-HIPS by a simple and efficient electrophilic substitution reaction was directed to enhance the hydrophilicity, morphology, permeability, and antifouling properties of HIPS membranes [23,24]. R-HIPS sulfonated asymmetric membranes’ general properties and performance in removing salts and dyes under low-pressure conditions and the effect of R-HIPS sulfonation degree on membrane fouling are discussed. Overall, this study has the potential to make a significant impact on both reducing plastic pollution and improving wastewater treatment systems through the use of recycled HIPS. With further research focused on the scalability as spiral-wound filtration modules and addressing potential challenges, sulfonated high-impact polystyrene membranes could emerge as a cost-effective and sustainable alternative for polystyrene recycling and wastewater treatment.

## 2. Materials and Methods

### 2.1. Chemicals

Recycled high-impact polystyrene (R-HIPS) was obtained from a Cole-Parmer tray. Ethyl acetate (99.5%, M TEDIA, Fairfield, CA, USA), chlorosulfonic acid (CSA, 99.0%), 1-2-dichloroethane (DCE, 99.8%), bovine serum albumin (BSA, 67 kDa), potassium phosphate di-basic (K_2_HPO_4_, 98%), magnesium sulfate (MgSO_4_, ≥97%), sodium sulfate (Na_2_SO_4_, ≥99%), Reactive Black 5, 1methyl-2-pyrrolidinone anhydrous (NMP, 99.5%), toluene (HPLC, 99.9%), methanol (MeOH, 99.8%), and phenolphthalein (ACS reagent) were all supplied by Sigma Aldrich S.A. de C.V. (Toluca, México) and used as received. Potassium phosphate monobasic (KH_2_PO_4_, 99.5%, Fluka chemical, GmbH, Buchs, Switzerland), 2-propanol (99.8%, J.T. Baker, Edo. de México, México), acetic acid 5% solution in water (commercial vinegar, @La Costeña, Yucatán, México), ethanol (EtOH, 96%, CTR Scientific, Nuevo León, México), sodium bicarbonate (NaHCO_3_, Arm & Harmmer, Church & Dwight, Ewing, NJ, USA), and sodium hydroxide (NaOH, J.T. Baker, 99.5%, Edo. de México, México) were also used as received.

### 2.2. R-HIPS Sulfonation

R-HIPS sulfonation was carried out by direct sulfonation in solution with chlorosulfonic acid (CSA) at two different concentrations (3 and 5%). In a typical sulfonation reaction, in a 500 mL three-neck flask with a mechanical stirrer, 10 g of R-HIPS was dissolved in 100 mL of DCE. Then, the homogeneous solution was cooled in an ice bath, and the required amount of chlorosulfonic acid was added dropwise, using an addition funnel to achieve the desired sulfonation degree (see Table 1). After this point, the sulfonation reaction was stirred at room temperature for 20 h. The resulting product was then gradually poured into ethanol while being stirred vigorously. The sulfonated R-HIPS was filtered, washed with EtOH, and dried in a vacuum oven at 60 °C for 24 h [25].

Sulfonation degree (SD) was determined by a titration method as follows: 100 mg of R-HIPS (in triplicate) was solubilized in 10 mL of a toluene/methanol mixture (90/10 *v*/*v*) and subsequently titrated with a 0.01 M NaOH/methanol solution, using phenolphthalein as an indicator. The SD value (%) was calculated by Equation (1) [26]:

(1) $$SD=\frac{104\times N_{NaOH}{\times V}_{NaOH}}{\left(w\times 1000)-(81\times N_{NaOH}\times V_{NaOH}\right)}$$

where $N_{NaOH}\;$ and $V_{NaOH}$ are the concentration (mol/L) and volume of methanolic NaOH solution (mL), and W is the R-HIPS weight (g). The numbers 104 and 81 are the molar mass of the styrene unit and sulfonic, –SO_3_H, groups, respectively.

### 2.3. Preparation of Asymmetric Membranes

The membranes were prepared by the phase inversion method using a 20% (*w*/*v*) doping solution in NMP. The polymer solution was cast on a glass support using a knife blade previously graduated at 33 µm, and it was immersed in the coagulation bath, which consisted of a mixture of commercial vinegar (acetic acid 5% solution in water)/isopropanol (15/85% *v*/*v*) with 0.12% NaHCO_3_ at room temperature, at 50% relative humidity. Finally, the membranes were stored in distilled water for further characterization. Based on the sulfonation degree (SD), the membranes were identified as R-HIPS (non-sulfonated), R-HIPS-3 (3% SD), and R-HIPS-5 (5% SD).

### 2.4. Characterization

A scanning electron microscope (SEM) (JEOL JSM-6360LV SEM, Tokyo, Japan) was used to obtain cross-sectional and surface micrographs of membrane samples previously coated with gold in a DENTON VACUUM DESK II equipment (Philadelphia, NJ, USA).

FTIR-ATR spectra of R-HIPS and sulfonated R-HIPS were recorded on a Nicolet 8700 FTIR (Waltham, MA, USA) in the 4000–650 cm^−1^ range. Contact angle measurements on R-HIPS membrane surface were determined on a Ramé-Hart 590 goniometer equipped (Succasunna, NJ, USA) with a high-resolution drop image software.

Water uptake was measured by immersing dry samples (1.5 cm × 1.5 cm) in water for 7 days at room temperature. The dry weight was determined after drying the samples at 60 °C for 24 h. The percentage of water content (WC %) was calculated with the following Equation (2).

(2) $$WC(\%)=\frac{W_{0}-W_{1}}{W_{0}}\times 100$$

where $W_{0}$ = is the membrane wet weight, and $W_{1}$ = is the membrane dry weight.

### 2.5. Membrane Performance Assessment

Membrane porosity (ɛ) was calculated from the difference between the bulk membrane volume (V_m_) and the polymer volume (V_p_). The porosity was calculated using Equation (3) [27].

(3) $$\varepsilon (\%)\;=\frac{V_{m}-V_{p}}{V_{m}}\times 100$$

where $V_{m}$ is the bulk membrane volume, and $V_{p}$ represents the polymer volume. $V_{m}$ was obtained by multiplying the membrane area by its thickness. The polymer volume $V_{p}$ was obtained by the ratio Wmρp, where $W_{m}$ is the membrane weight, and $\rho_{p}$ is the polymer’s density.

The mean pore radius was calculated using the Guerout–Elfort–Ferry equation (Equation (4)).

(4) $$r_{m}\;=\;\sqrt{\frac{\left(2.9\;-1.75\varepsilon \right)8\eta \delta_{0}Q}{\varepsilon A△P}}$$

where η is the pure water viscosity (8.9 × 10^−4^ Pa s), Q is the pure water volume permeated per unit of time (mm^3^s^−1^), A is the effective area of the membrane (mm^2^), $\delta_{0}$ is the thickness of the membrane (mm), and ∆P is the transmembrane pressure (Pa).

### 2.6. Membrane Filtration Experiments

Membrane filtration experiments were conducted using a dead-end filtration module (Sterlitech HP4750 Stirred Cell) with magnetic stirring at 500 rpm, using an effective membrane area of 14.6 cm^2^. Initially, the membrane was compacted at 30 psi for 1 h; after this time, 100 mL of deionized pure water was permeated through the membrane to determine an initial flux J_w1_, followed by three 100 mL filtration cycles of a 1.0 mg/mL BSA solution to obtain J_BSA_. Finally, the membrane was cleaned with distilled water for 30 min, and the first permeate after cleaning was taken as water flux J_w2_. This procedure allowed for the assessment of the flux recuperation ratio (FRR) and BSA flux recovery ratio (Rr), which were calculated using Equations (5) and (6) [27].

(5) $$FRR\left(\%\right)=\frac{J_{w2}}{J_{w1}}\times \;100$$

(6) $$Rr\left(\%\right)\;=\left(\frac{J_{w2\;}-\;J_{BSA}}{J_{w1}}\right)\;\times \;100$$

Moreover, total flux loss caused by the total fouling (Rt) and percentage of irreversible flux loss caused by irreversible fouling (Rir) were calculated using Equations (7) and (8), respectively.

(7) $$Rt\left(\%\right)\;=\left(1\;-\frac{J_{BSA}}{J_{w1}}\right)\;\times \;100$$

(8) $$Rir\left(\%\right)\;=\left(\frac{J_{w1\;}-\;J_{w2}}{J_{w1}}\right)\;\times 100$$

### 2.7. Salt and Dye Rejection Experiments

The salt and dye rejection methodologies were conducted in a similar way to the one described for the BSA filtration experiment in the Sterlitech HP4750 Stirred Cell (Auburn, WA, USA). For salt rejection, 100 mL of 0.5 mg/mL MgSO_4_ solution or Na_2_SO_4_ solution (500 ppm) was permeated through R-HIPS membranes at 30 psi, whereas for dye rejection, a 100 mL solution containing 0.05 mg/mL of Reactive Black 5 (50 ppm) was used. The permeation and retention fluxes of salt solution were calculated using Equations (9) and (10):

(9) $$J\;=\frac{V}{A\cdot t}$$

(10) $$R\left(\%\right)=\left(1-\frac{C_{p}}{C_{f}}\right)\times 100$$

where $C_{p}$ is the concentration of salt or dye in the permeate, and $C_{f}$ is the salt or dye concentration in the feed. Salt rejection was measured using a conductometer. In the dye rejection experiments, concentrations were determined using a UV-Vis spectrometer at 595 nm.

## 3. Results and Discussion

The introduction of sulfonic groups (-SO_3_H) into the polymer chain can significantly enhance important properties, such as hydrophilicity, permeability, morphology, and antifouling capacity [28,29]. In this study, the primary goal in modifying R-HIPS membranes was to make them less susceptible to fouling by changing them from a hydrophobic to hydrophilic nature through varying concentrations of -SO_3_H. The experimental sulfonation degrees (SDs) of R-HIPS membranes ranged from 0% to 5.9%, closely aligning with the theoretical SD, as can be seen in Table 1. Initial experimental tests for membrane fabrication were conducted using a higher SD (10, 15, and 20%) of recycled HIPS. However, at these sulfonation levels, HIPS becomes partially soluble in water and alcohols, which complicates both the membrane formation process and their application in water treatment.

**Scheme 1:**
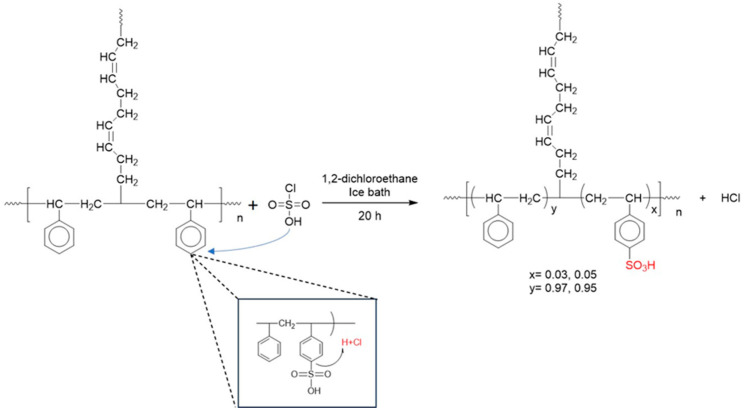
Proposed reaction mechanism for HIPS sulfonation with CSA.

The reaction mechanism for HIPS sulfonation with chlorosulfonic acid is a typical electrophilic aromatic substitution, which introduces a sulfonic acid group on the benzene rings of the polystyrene fragment, as depicted in Scheme 1 [25].

**Figure 1:**
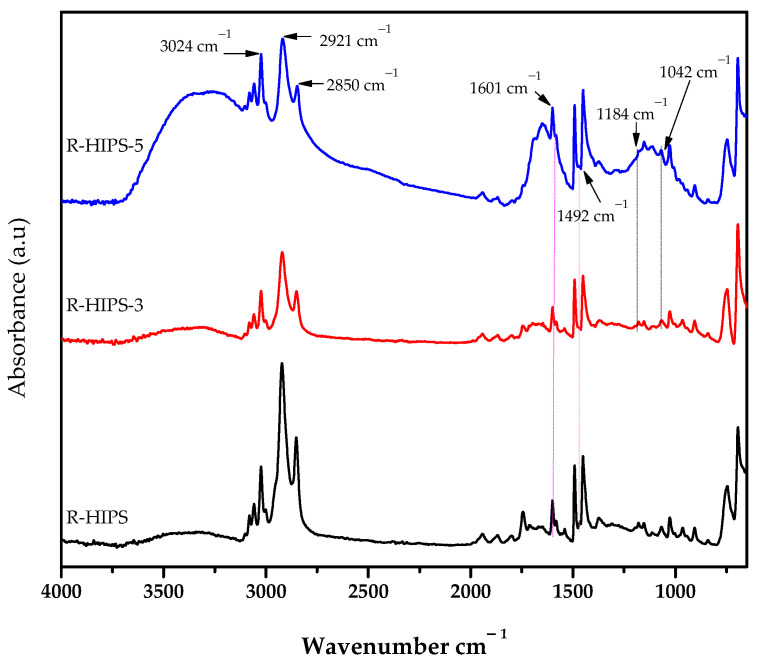
ATR-FTIR spectra of R-HIPS and sulfonated R-HIPS.

FTIR spectroscopy was employed to verify the chemical structures of R-HIPS and sulfonated R-HIPS-3 and R-HIPS-5. Figure 1 shows that the aromatic C-H stretching vibrations appeared at 3082, 3058, and 3024 cm^−1^. Additionally, the peaks centered at 2921 cm^−1^ and 2850 cm^−1^ corresponded to the symmetric and asymmetric stretching of C-H bonds of vinyl groups, respectively [14]. The spectra also displayed vibrations at 1601 cm^−1^ and 1492 cm^−1^, which were attributed to aromatic C=C stretching. Furthermore, all spectra exhibited characteristic polybutadiene signals between 994 and 720 cm^−^^1^, attributed to the deformation bands of vinyl-1,2, *trans*-1,4, and cis-1,4 configurations [30]. The peaks around 1184 and 1042 cm^−^^1^, assigned to the antisymmetric and symmetric vibrations of the SO_3_^−^ group [31], may overlap with absorption peaks from additives in R-HIPS, complicating the identification of those signals. Moreover, a broad peak between 3100 and 3650 cm^−1^ was observed, corresponding to O–H stretching vibrations from hydrogen bonds. This peak was intensified with an increasing sulfonation degree, indicating that higher sulfonation enhances polymer hydrophilicity.

Changes in the hydrophobic character of R-HIPS due to sulfonation could modify the thermodynamic equilibrium for the R-HIPS solution behavior between the polymer/solvent/non-solvent system during the membrane formation process [25]. As reported in the literature, R-HIPS membranes with macrovoids in their structure and larger cavities in the sublayer are obtained using deionized water as non-solvent in asymmetric membrane preparation, due to an instantaneous demixing [14,15,19,20]. As a result, the formation of macrovoids and larger cavities can indeed compromise the mechanical stability of R-HIPS membranes. Thus, it is important to consider alternative non-solvents or look into adjusting the casting conditions to obtain delayed demixing processes to avoid the formation of macrovoids and large cavities. In this case, after several tests, commercial vinegar/isopropanol (15/85% *v*/*v*) containing 0.12% NaHCO_3_ was selected as the coagulation bath to reduce macropore formation in the membrane’s cross-section and promote the formation of the asymmetric membrane with a sponge-like porous structure. This formulation closely follows those reported by Yam-Cervantes et al. for asymmetric membranes preparation from sulfonated polymers [24]. Additionally, this coagulation bath was chosen because it was not favorable to use water as a non-solvent for sulfonated membranes formation.

### 3.1. Membrane Morphology

**Figure 2:**
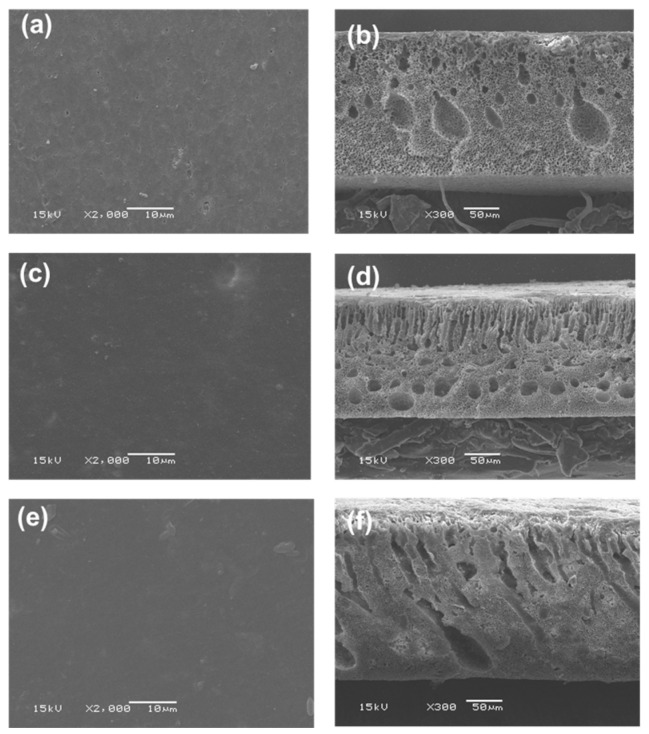
SEM images of surface and cross section at 2000× and 300× of (**a**,**b**) R-HIPS, (**c**,**d**) R-HIPS-3, and (**e**,**f**) R-HIPS-5, respectively.

Figure 2 displays the SEM images of R-HIPS, R-HIPS-3, and R-HIPS-5. The sublayer surfaces of R-HIPS (Figure 2a) exhibited micro-sized pores, while the sulfonated membranes R-HIPS-3 (Figure 2c) and R-HIPS-5 (Figure 2e) revealed completely dense surfaces. The cross-section morphologies of R-HIPS and sulfonated R-HIPS-3 and R-HIPS-5 revealed a sponge-like matrix with rounded and elongated macropores (Figure 2b,d,f), with this trend becoming more pronounced as the sulfonation degree increased. This phenomenon can be attributed to a delayed demixing process in the coagulation bath. This effect is due to hydrogen bonding interactions between sulfonic acid groups and water molecules present in the vinegar. According to Feng et al. [32], the presence of hydrogen bonding increases dope viscosity, which slows down the convection and diffusion processes between the solvent and nonsolvent during membrane formation. The increased viscosity can lead to a reduced droplet growth rate, resulting in a smaller pore size distribution within the membrane [33]. The morphology of sulfonated R-HIPS allows for better stability in the as-prepared membranes.

Table 2 presents the % porosity (ɛ) and pore radius (r_m_) values for R-HIPS and sulfonated R-HIPS membranes prepared in this work and their comparison with some recycled HIPS membranes reported in literature [15,21]. This study confirmed that the pore radius decreased slightly from 4.0 nm to 3.3 nm with an increasing sulfonation degree (Table 2), which also led to a reduction in membrane porosity (ɛ). However, no significant decrease in the finger-like structures within the cross-section of R-HIPS sulfonated membranes was observed, which may be attributed to NaHCO_3_ addition that weakened the hydrogen bonding interactions between -SO_3_H groups and water molecules [34]. The membrane porosity (ɛ), calculated using the dry–wet weight method, decreased from 75 for R-HIPS to 63.9% in R-HIPS-3. Increasing sulfonation for R-HIPS-5 showed a slight porosity increase (67.0%) in comparison with R-HIPS-3, which was attributed to the presence of elongated macropores. As shown in Table 2, for membranes from recycled HIPS reported in literature, termed HIPS-R [15], 30% and 35% [21] exhibited higher pore radii that led to membranes for ultrafiltration and microfiltration, resulting in increased flux and lower solutes retention. Thus, this larger pore size compromised the membrane selectivity and overall performance. The increase in pore size is attributed to the instantaneous demixing formation that occurs during the fabrication process when water is used as a non-solvent.

### 3.2. Hydrophilicity

**Figure 3:**
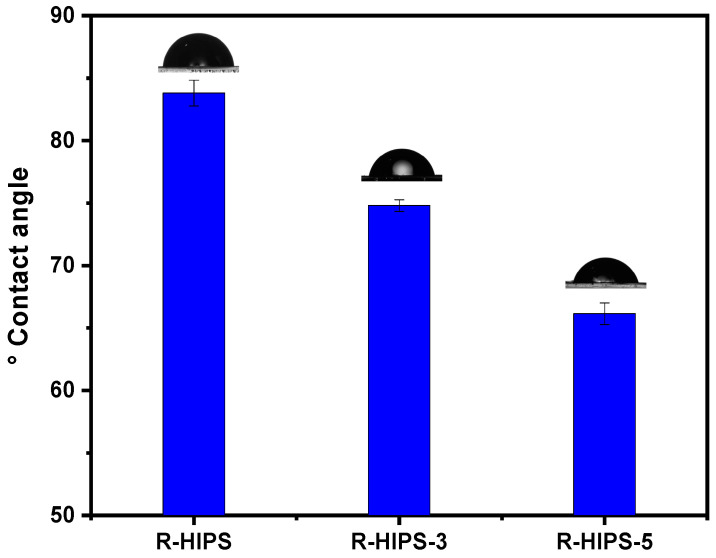
Water contact angles of R-HIPS, R-HIPS-**3**, and R-HIPS-5 membranes.

Water contact angle (WCA) was determined to verify changes in hydrophilicity for R-HIPS membrane surfaces (Figure 3). The contact angles decreased from 83.8° to 66.2° with an increasing sulfonation degree from 0 to 5%. This reduction was due to the presence of strongly hydrophilic -SO_3_H groups, which enhanced membrane surface interaction with water, facilitating the filling of air voids and enhancing overall wetting [35]. In general, porosity, pore size, and hydrophilicity are key factors that determine water permeability and solute rejection properties of membranes [36]. Higher pore size generally allows for increased flux, but it may compromise the membrane ability to reject solutes. On the other hand, a significant challenge in filtration processes is membrane fouling, which occurs when unwanted materials accumulate on the membrane surface or within its pores, due to, among other factors, membrane hydrophobicity. Fouling not only reduces filtering efficiency but also decreases the average membrane life and increases operating costs [37]. Thus, these properties (porosity and hydrophilicity) are crucial for optimizing membrane performance in applications such as water treatment for dye and salt elimination and separation processes, where organic suspended matter is present.

Furthermore, HIPS, R-HIPS-3, and R-HIPS-5 membrane swelling tests support the results found by contact angle measurements. HIPS, R-HIPS-3, and R-HIPS-5 showed a higher water uptake as SD increased (see Table 2). After immersion for 7 days in water, R-HIPS and R-HIPS-3 showed water uptake values of 2.2% and 3.6%, respectively, while R-HIPS-5 exhibited a significantly higher uptake of 10.6%, following a similar trend to the contact angle measurements. Moreover, weight losses after immersion were 1.2, 3.2, and 6.0% for R-HIPS, R-HIPS-3, and R-HIPS-5 membranes, respectively (Table 2).

### 3.3. Membrane Antifouling Properties and Water Permeability

**Figure 4:**
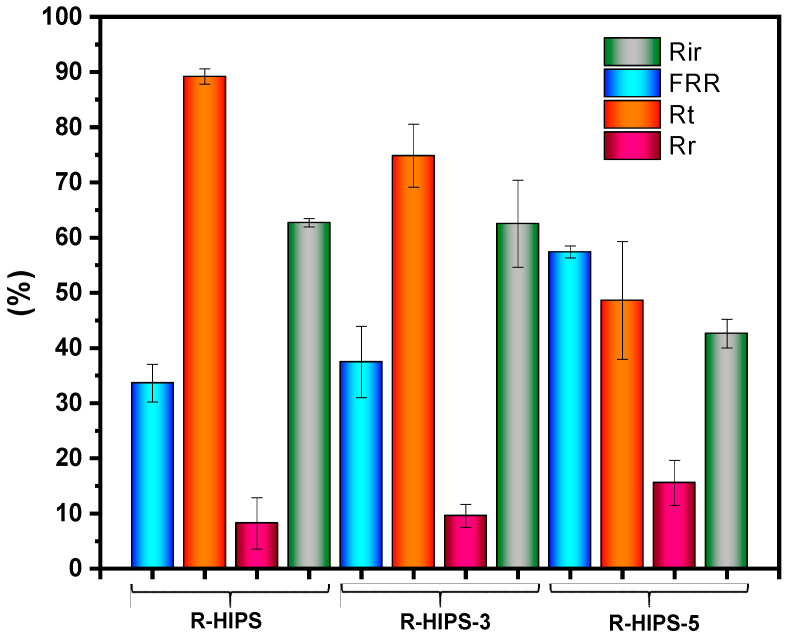
Antifouling performances observed with BSA solutions of R-HIPS, R-HIPS-3, and R-HIPS-5 membranes.

The antifouling performances of R-HIPS, R-HIPS-3, and R-HIPS-5 membranes were evaluated by dynamic permeation experiments using a 1000 ppm BSA solution. The flux recovery ratio (FRR %), irreversible fouling (Rir), recovery capacity (Rr), and the total fouling (R_t_) are presented in Figure 4. The flux recovery ratios (FRR) of sulfonated R-HIPS-5 and R-HIPS-3 membranes were greater than that of R-HIPS, indicating that increased hydrophilicity enhanced the antifouling properties of the membranes. The reversible fouling ratios (Rr), which refers to the temporary adsorption of molecules or particles onto a membrane surface, were 8.2%, 9.6%, and 15.6% for R-HIPS, R-HIPS-3, and R-HIPS-5, respectively. In contrast, the irreversible fouling ratio (Rir), which occurs when foulants such as proteins, oils, or particulates become permanently fixed onto the pore surfaces of a membrane [38], was lower in R-HIPS-5 membrane, following the same tendency (R-HIPS > R-HIPS-3 > R-HIPS-5) as the total fouling (R_t_), which indicates that increasing sulfonation reduces the fouling in R-HIPS membranes. The notably lower Rr values compared to Rir suggest that particles tend to adhere more readily to the pore surfaces in the non-sulfonated R-HIPS membrane, compared to R-HIPS.

Also, in the dynamic permeation experiments, the R-HIPS membrane exhibited a 6.0 L/m^2^h water flux, while the sulfonated membranes, R-HIPS-3 and R-HIPS-5, showed reduced fluxes at 4.3 L/m^2^h and 3.1 L/m^2^h, respectively. This reduction in water flux is consistent with the membrane porosity results, since a larger degree of sulfonation showed a reduced porosity.

### 3.4. Separation Performance

**Figure 5:**
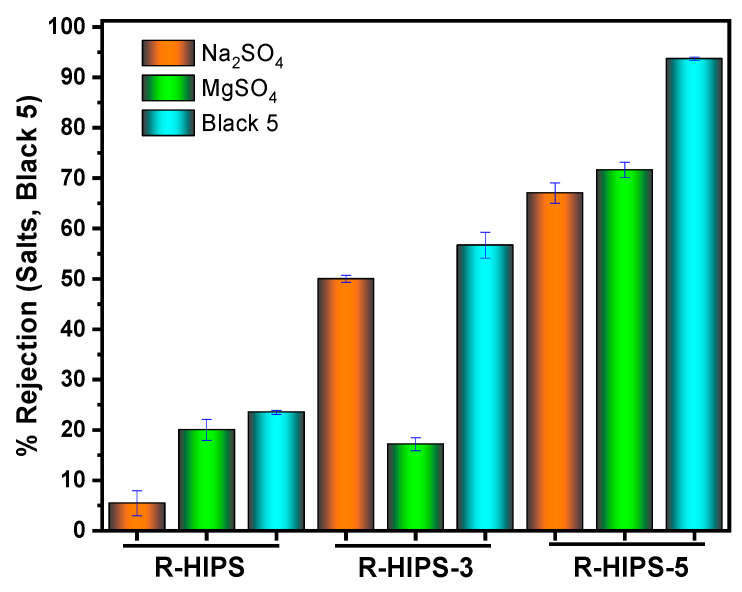
Salts and dye rejection performances of R-HIPS, R-HIPS-3, R-HIPS-5 membranes.

To evaluate the efficiency of the recycled HIPS membranes in inorganic and organic separation process, three different solutes were used: magnesium sulphate (MgSO_4_, a univalent salt), sodium sulphate (Na_2_SO_4_, a divalent salt), and Reactive Black 5 dye. Figure 5 summarizes the separation performances of R-HIPS and sulfonated R-HIPS membranes, highlighting their effectiveness in filtering these selected solutes. The dye rejection in R-HIPS membranes correlated with a morphology of decreasing pore size, increasing hydrophilicity, and an enhanced negative charge in sulfonated membranes (see Figure 5). R-HIPS membrane exhibited the lowest rejection of Black 5 dye at 5.5%, which can be attributed to the micro-sized pores present on its surface. In contrast, R-HIPS-3 (57% dye rejection) and R-HIPS-5 membranes, which had the smallest pore size (3.3 nm), almost completely blocked the dye, achieving a 94% dye rejection. Due to the fact that R-HIPS sulfonated membranes and the dye have similar charges, the electrostatic repulsion between them can prevent the dye from permeating through the membrane [39]. Additionally, this repulsion helps to minimize the dye adsorption onto the membrane surface, which can contribute to reducing membrane fouling.

Regarding salts rejection, the R-HIPS membranes showed a tendency where R-HIPS-5 exhibited the highest rejection, followed by R-HIPS-3 and R-HIPS. This order can be attributed to pore size and surface properties. The smaller pore size of the R-HIPS-5 membrane contributed to its enhanced ability to reject divalent and monovalent salts, as smaller pores can physically block larger ions from passing through them. Additionally, the higher negative charge from the sulfonic acid groups enhanced electrostatic repulsion, particularly against positively charged ions, improving rejection efficiency [35].

Moreover, more hydrophilic membranes tend to have better antifouling properties, and they can interact more effectively with water, facilitating selective ion transport while reducing the passage of salts. Thus, the combination of smaller pore size and increased negative charge is crucial for the superior performance of R-HIPS-5 in rejecting salts.

## 4. Conclusions

It was found that chlorosulfonation in solution is an efficient method to prepare R-HIPS sulfonated material for membrane preparation. Sulfonated recycled HIPS asymmetric membranes were prepared using a phase inversion method, with a coagulation bath of commercial vinegar/isopropanol (15/85% *v*/*v*) and 0.12% NaHCO_3_. This process resulted in the formation of asymmetric membranes, with pore sizes ranging from 4.0 to 3.3 nm and porosities between 75.0% and 63.9%, attributed to a delayed demixing process, particularly for R-HIPS sulfonated membranes. The degree of sulfonation significantly impacted the membrane’s morphology, hydrophilicity, antifouling properties, and particle rejection capabilities for substances such as MgSO_4_, Na_2_SO_4_, and Black 5 dye. As the sulfonation degree increased, R-HIPS, R-HIPS-3, and R-HIPS-5 membranes exhibited a decrease in pore size and porosity, with a slight reduction in water permeation flux, dropping from 6.0 L/m^2^h in the R-HIPS membrane to 3.1 L/m^2^h in the R-HIPS-5. Notably, higher sulfonation levels enhanced hydrophilicity, leading to improved antifouling properties and separation performance. In particular, R-HIPS-5 membrane demonstrated promising results, achieving a separation efficiency of 94% for Black 5, 72% for MgSO_4_, and 67% for Na_2_SO_4_, with a high flux recovery ratio of 58%. These results are attributed to a smaller pore size and increased sulfonic acid group concentration in R-HIPS-3 and particularly R-HIPS-5 membranes, which enhanced their rejection efficiency.

Given the feasibility of producing flat membranes and their separation properties, these membranes could be useful for the production of spiral-wound modules. Scaling up could enhance their separation efficiency in large-scale wastewater treatment processes for the removal of dyes from water industrial stream and for industrial product purification applications. Recycling materials such as HIPS waste offers an alternative way to reduce plastic waste and reintroduce it into the circular economy using membranes for water purification, increasing its value.

## Figures and Tables

**Table 1:** Amounts of each reactant used based on 10 g of sulfonated R-HIPS.

Membranes	Theoretical SD (%)	1,2, Dichloroethne (mL)	Chlorosulfonic Acid (mL)	Experimental SD (%)
R-HIPS-3	3	100	0.19	2.7
R-HIPS-5	5	100	0.33	5.9

**Table 2:** Water contact angle, water uptake, weight loss, % porosity, radius pore size, and flux of R-HIPS membranes.

Membrane	Contac Angle (^o^)	Water Uptake (%)	Weight Loss (%)	Porosity (ɛ) (%)	r_m_ (nm)	Flux (L/m^2^h)
R-HIPS	83.8 ± 1.034	2.2 ± 0.5	1.2± 0.33	75.0	4.0 ± 0.32	6.0 ± 0.84
R-HIPS-3	74.8 ± 0.26	3.6 ± 2.9	3.2 ± 0.61	63.9	3.8 ± 0.08	4.3 ± 0.13
R-HIPS-5	66.1 ± 0.87	10.6 ± 0.4	6.0 ± 0.95	67.0	3.3 ± 0.67	3.1 ± 1.02
HIPS-R [15]	81.7 ± 3.42	51.7 ± 5.5	-	52.3	32.4 ± 1.12	180.6 ± 0.71
30 wt% [21]	83.0	-	-	62.5	50–500	8.5
35 wt% [21]	91.0	-	-	61.6	50–250	8.3

## Data Availability

The original contributions presented in the study are included in the article, further inquiries can be directed to the corresponding author.
